# Vertical integration of biochemistry: The interdisciplinary spiral curriculum in the Brandenburg reformed medical study programme

**DOI:** 10.3205/zma001857

**Published:** 2026-06-15

**Authors:** Jenny Engelmann, Julia Schendzielorz, Fabian Otte, Alexander Bertram, Meike Hoffmeister, Stefanie Oess

**Affiliations:** 1 Brandenburg Medical School Theodor Fontane (MHB), Institute of Biochemistry, Neuruppin, Germany; 2 Faculty of Health Sciences, Joint Faculty of the University of Potsdam, Brandenburg Medical School Theodor Fontane and Brandenburg Technical University Cottbus-Senftenberg, Neuruppin, Germany; 3 Brandenburg Medical School Theodor Fontane (MHB), Center for Curriculum Development and Educational Research (ZSAW-BB), Neuruppin, Germany

**Keywords:** medical education, curriculum, teaching, biochemistry

## Abstract

**Objective:**

A key recommendation for further improvement of undergraduate medical education is vertical integration, in particular the close connection of basic medical science and clinical learning content. The project described here aimed to develop and implement an interdisciplinary spiral curriculum for biochemistry, which has its own subject systematics, is integrated into the organ- and topic-related modules of the Brandenburg reformed medical study programme and exploits learning theory-based benefits of vertical integration. This project report provides a specific implementation example for practitioners.

**Methodology:**

Curriculum development took place in an interdisciplinary process. Learning success was analysed comparatively using the Progress Test Medicine, and student perception was evaluated using a questionnaire.

**Results:**

The spiral curriculum for biochemistry is fully integrated into a Z-curriculum, both across the entire length of the study programme as well as at the level of modules, modules’ learning objectives, weekly topics and interdisciplinary classes. Exemplary mapping with the NKLM and the national catalogue of exam-relevant topics, comparable learning success with other universities and student evaluations confirm the intended subject systematics, the high degree of vertical integration and its positive evaluation, e.g. in terms of relevance of learning content and deep learning. According to level 9 of Harden's integration ladder, the biochemical subject identity has been retained, but subject autonomy has been largely abandoned.

**Conclusion:**

The spiral curriculum for biochemistry implements key recommendations for further improvement of undergraduate medical education, demonstrates the successful application of the principles of vertical integration and confirms the opportunities associated with its use.

## Zusammenfassung

**Zielsetzung:**

Zentrale Forderung für die Weiterentwicklung des Medizinstudiums ist die vertikale Integration, insbesondere die enge Verknüpfung von grundlagenwissenschaftlichen und klinischen Lerninhalten. Im beschriebenen Projekt sollte ein interdisziplinäres Spiralcurriculum Biochemie mit eigener Fachsystematik entwickelt und implementiert werden, das in die organ- und themenbezogenen Module des Brandenburgischen Modellstudiengangs Medizin integriert ist und die lerntheoretischen Vorteile der vertikalen Integration nutzt. Der Projektbericht bietet ein konkretes Umsetzungsbeispiel für Praktiker*innen.

**Methodik:**

Die Entwicklung erfolgte in einem interdisziplinären Prozess. Der Lernerfolg wurde mit Hilfe des Progress Test Medizin vergleichend analysiert und die studentische Bewertung mittels eines Fragebogens evaluiert.

**Ergebnisse:**

Das Spiralcurriculum Biochemie ist sowohl über den gesamten Studienverlauf als auch auf Ebene der Module, Modullernziele, Wochenthemen und interdisziplinären Lehrveranstaltungen vollständig in ein Z-Curriculum integriert. Ein exemplarisches Mapping mit NKLM und Gegenstandskatalog, der mit anderen Universitäten vergleichbare Lernerfolg und die Evaluation der Studierenden belegen die angestrebte Fachsystematik, die hochgradige vertikale Integration und deren positive Bewertung, z. B. in Bezug auf Relevanz und Förderung des Verständnisses der Lerninhalte. Gemäß Stufe 9 der Integrationsleiter nach Harden ist die biochemische Fachidentität erhalten, die Fachautonomie hingegen weitgehend aufgegeben.

**Schlussfolgerung:**

Das Spiralcurriculum Biochemie setzt zentrale Forderungen zur Weiterentwicklung des Medizinstudiums um, demonstriert die erfolgreiche Anwendung der Prinzipien der vertikalen Integration und bestätigt die mit ihrem Einsatz verbundenen Chancen.

## 1. Introduction

### 1.1. Background

**Figure 1:**
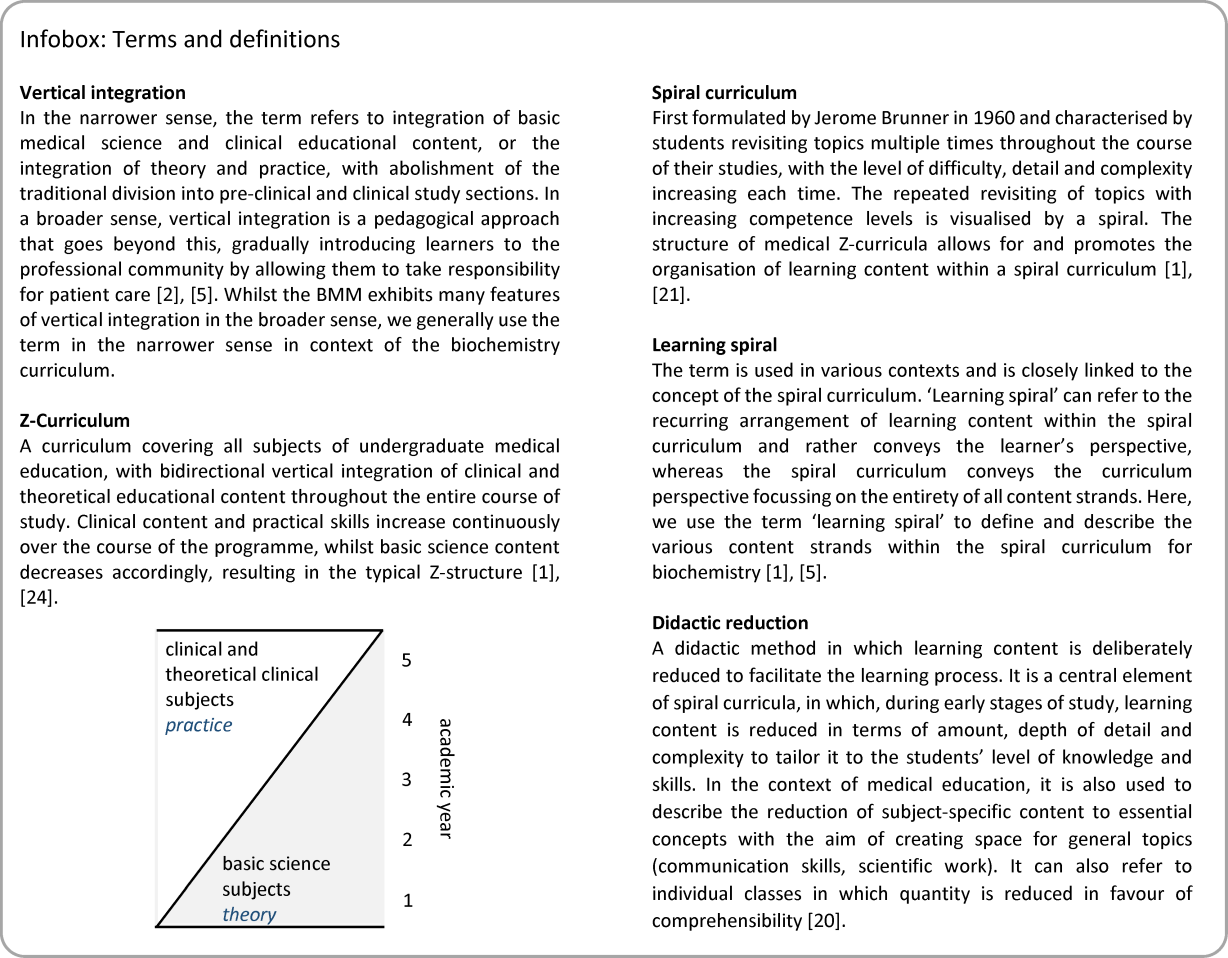
Key terms, their definitions and use in this project report

Key recommendations for further improvement of undergraduate medical education are a modular, interdisciplinary course structure that abandons the separation between preclinical and clinical study sections and the intertwining of theoretical and practical content throughout the entire length of the study programme [1]. For basic medical sciences like biochemistry, the development of such interdisciplinary Z-curricula, characterised by horizontal integration with other basic science subjects and vertical integration with clinical subjects, is challenging (see figure 1 (Fig. 1)). In particular, this challenge lies in arranging the learning content according to the subject’s internal logic while at the same time integrating it into organ- or topic-related modules.

With this project report, we provide a specific example of how these recommendations can be implemented, applying learning theory-derived principles to the design of vertically integrated Z-curricula. We address practitioners with responsibility for teaching, teaching coordination and curriculum development in order to demonstrate feasibility and opportunities, promote discussion within and across faculties, and support current developments in medical education.

### 1.2. The Brandenburg reformed medical study programme

The Brandenburg reformed medical study programme (*Brandenburgischer Modellstudiengang Medizin, BMM*) is a reformed or model degree programme in accordance with § 41 of the German Medical Licensing Regulation (*Ärztliche Approbationsordnung, ÄApprO, 2002*). Structure, content and teaching philosophy are characterised by comprehensive vertical integration [2]: The BMM has a Z-curriculum and is organised in modules. At the beginning of the programme, modules focussing on basic medical sciences incorporate clinical content, while towards the end of the programme, basic science content is imbedded in clinically defined modules. From the first year onwards, students are involved in patient care with gradually increasing responsibility (see figure 1 (Fig. 1)). The curriculum is competency-based; teaching and examination follow centrally defined cognitive, psychomotor/communicative as well as affective learning objectives. Problem-based learning (PBL) is the central teaching/learning format and structures the curriculum: the week typically begins and ends with a PBL case tailored to the topic of the week. Within this PBL frame, two interdisciplinary seminars (IDS), a practical class and an exercise in diagnostics and therapy (EDT) are offered to support students in achieving their self-set learning goals. An IDS is taught jointly by two teachers from two disciplines. Summative assessments take place at the end of each semester across all modules and use various examination formats, in particular multiple choice (MC) questions, objective structured clinical/practical examination (OSCE/OSPE) parcours and oral-practical examinations. In MC exams, the number of questions per subject and learning objective is determined by a centrally defined blueprint [3], [4].

### 1.3. Learning theory basis of vertical integration

The integration of basic medical sciences, especially vertical integration, is associated with various advantages derived from learning theory models and research [2], [5]: Through the clinical context, students recognise the relevance of the basic science content and thus the purpose of learning, which, according to adult learning theory [6], [7], motivates them to engage with the topic. Another advantage relates to the way in which knowledge is stored and retrieved in memory and is supported by findings in cognitive psychology [8], [9]. These indicate that knowledge is most effective when its organisation matches the way in which the knowledge is to be used. In the context of medical education, this means that integrating basic science content with clinical examples and explicitly linking the concepts of both areas promotes knowledge retrieval, long-term retention and a deeper understanding [2], [5]. Finally, by repeatedly applying basic sciences to explain various clinical scenarios, vertical integration promotes not only deep learning of the basic sciences but also allows students to derive broader basic science concepts and thus facilitates transfer of basic science knowledge [5].

An inevitable consequence of integrating basic sciences into clinical contexts is the distribution across many relatively small units, whose arrangement is partly determined by the clinical discipline. This carries the risk that teaching may not be sufficiently systematic or in line with the internal logic of the subject, preventing students from recognising broader, subject-inherent connections and the overall conceptual picture [2], [10].

### 1.4 Objectives of the project presented here

We aimed to develop an interdisciplinary spiral curriculum for biochemistry that is fully integrated into the modules of the BMM and has its own subject systematics and internal logic. Integration should not only promote an understanding of clinical relevance, but also deepen the comprehension of biochemical subject knowledge and subject-inherent connections. Regarding subject systematics, it should be provided that, while applying didactic reduction, the subject is covered appropriately in terms of breadth and depth. At the same time, through the appropriate selection and arrangement of content units in a learning spiral, students should first work through simpler, less complex content and build on this knowledge in the course of their studies, deepening and expanding it (see figure 1 (Fig. 1)).

## 2. Project description

### 2.1. Conditions and procedure

The development of the spiral curriculum for biochemistry commenced with the appointment of the professor of biochemistry in the winter semester (WS) 17/18. At that time, approximately 150 students were enrolled in the BMM, spread across semesters 2, 4 and 6. The overall modular structure of the BMM had been developed and documented in the study regulations. The modules’ learning objectives (including those with biochemical content) and the classes with biochemical participation were defined for semesters 1-6.

The spiral curriculum for biochemistry was developed in an interdisciplinary process involving professor and staff of the Institute of Biochemistry, the department for curriculum development (meanwhile ZSAW-BB), colleagues from basic, clinical and theoretical clinical subjects, and BMM students. The existing learning objectives with biochemical content and the assignment of biochemistry to classes in semesters 1-6 were revised in WS 17/18. The development for semesters 7-10 followed successively until the summer semester (SS) 19. Interdisciplinary discussions took place both formally during regularly held module planning conferences and in informal exchanges. All development results were confirmed by the central study committee.

### 2.2. Spiral curriculum for biochemistry

#### 2.2.1. Extent, distribution and formats of biochemistry classes

**Figure 2:**
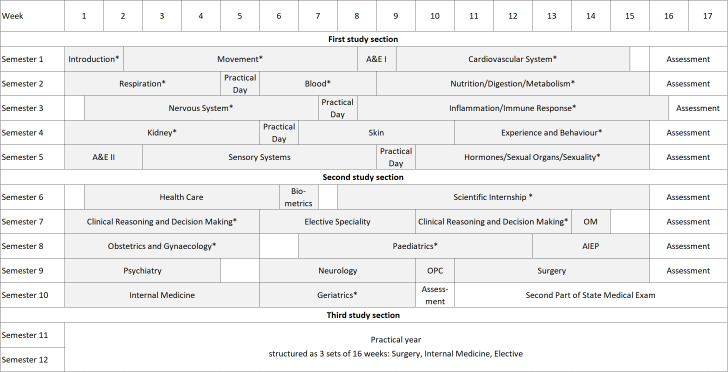
Representation of the subject of biochemistry within the BMM Modules in which the subject of biochemistry is involved are marked with an asterisk *. Presentation according to the BMM study regulations in the version dated 21 July 2022. AIEP: Anaesthesiology, intensive care, emergency medicine, pain therapy; A&E: Acute and emergency care; OM: Occupational medicine; OPC: Elective outpatient primary care

**Figure 3:**
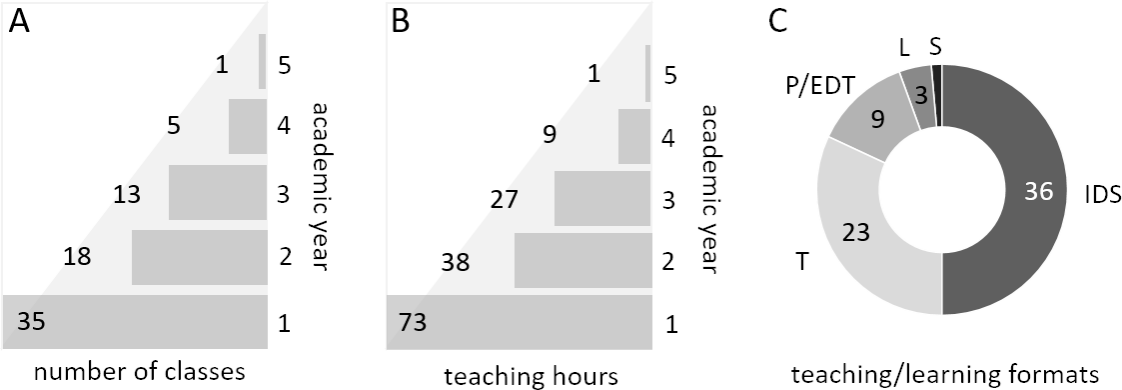
Biochemistry Z-curriculum in the BMM A Distribution of classes taught by or with the participation of biochemistry over academic years one to five of the BMM. B Number of teaching hours (45 min) of the classes shown in A. C Distribution of classes across the teaching/learning formats interdisciplinary seminar (IDS), tutorial (T), practical class (P), exercise in diagnostics and therapy (EDT), lecture (L) and seminar (S)

The spiral curriculum for biochemistry covers semesters 1-10 and biochemistry is represented in 16 of the 30 modules of the BMM (see figure 2 (Fig. 2)). The spiral curriculum for biochemistry comprises 72 individual classes with a total of 148 teaching hours (TH; 45 minutes each). Of a total of 1513 TH of module-dependent, subject-specific teaching (excluding scientific internship and hospital placement), 9.8% are therefore taught by or with the involvement of biochemistry. In line with the principle of the Z-curriculum, the majority of these classes lie in the 1^st^ and 2^nd^ academic year (see figure 3 A/B (Fig. 3)). Half of the biochemistry classes are IDS, while the other half is comprised of tutorials, practical classes, EDTs, lectures, and seminars (see figure 3 C (Fig. 3)). Of the IDS, 5 (14%) take place in horizontal integration with the basic medical science subjects anatomy and physiology, while the vast majority (31; 86%) are vertically integrated with clinical and theoretical clinical subjects. Over the course of the study programme, biochemistry cooperates with 16 different disciplines in the IDS format.

#### 2.2.2. Learning spirals of biochemical topics

In the spiral curriculum for biochemistry, the biochemical topics are distributed over a total of 23 learning spirals (see attachment 1 ). The individual learning spirals correspond to traditional biochemical themes developed inductively by staff of the Institute of Biochemistry from two standard textbooks [11], [12]. All learning spirals are addressed in at least three different modules and relate to four or more modules’ learning objectives.

#### 2.2.3. Detailed description of the learning spiral nucleic acids

The learning spiral nucleic acids spans semesters 1-10 and is integrated into nine different modules (see attachment 2 ). It is divided into 24 biochemical content units, which are simpler and less complex at the beginning of the programme and become increasingly detailed and extensive as the programme progresses. All content units address one or more of the centrally defined modules’ learning objectives. These are either explicitly phrased as subject-specific learning objectives focused on the topic of nucleic acids or integrate the nucleic acid aspect into an organ- or clinically-oriented learning objective. 18 of the content units are directly related to the weekly topic or PBL case. For these content units, the IDS is the most frequently used teaching format and the main focus is to establish interdisciplinary connections between nucleic acids and the weekly topic. Content units that are not directly related to the PBL case or weekly topic are mostly implemented in a flipped classroom format. Students can work on the asynchronous part at a time of their choosing using the learning platform (LP). The synchronous part takes place as a tutorial (T) and is not assigned to a specific module week. On the one hand, the content addressed in the LP/T teaching format is more focussed on foundational knowledge; on the other hand, specific cross-module longitudinal connections within the spiral curriculum are established explicitly.

All biochemical content units and module’s learning objectives are related to the contents of the National Competence Based Catalogue of Learning Objectives for Undergraduate Medical Education (*Nationaler Kompetenzbasierter Lernzielkatalog Medizin; NKLM*) [https://nklm.de/zend/menu] and the catalogue of exam-relevant topics (*Gegenstandskatalog; GK*) “Chemistry for Physicians and Biochemistry/Molecular Biology” [13]. They are almost exclusively assigned to chapters 12, 19.1 and 19.2 of the GK, which deal explicitly with nucleic acids and the storage, transmission and expression of genetic information, as well as subchapters in which these aspects are integrated into other subject areas (e.g. erythropoiesis). In addition, a few topics listed in the GK “Biology for Physicians” are taught by biochemistry. In return, a few topics that are traditionally covered by biochemistry fall under the responsibility of other subjects in the BMM, e.g. the topic “antibiotic mode of action of sulfonamides” (GK 19.1.1) is assigned to pharmacology.

#### 2.2.4. Intersection of several biochemical learning spirals in the IDS Joint Pain

**Figure 4:**
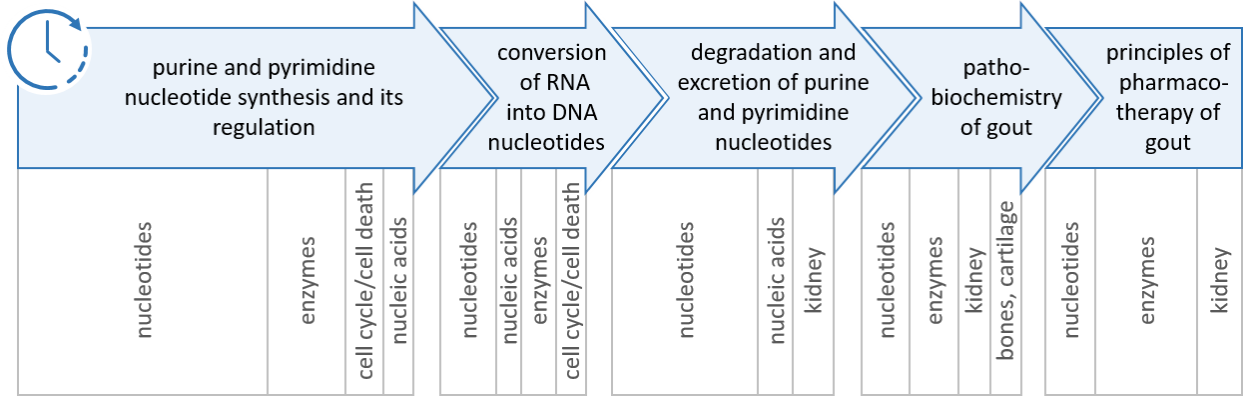
Biochemical learning spirals addressed in the IDS joint pain Schematic representation of the structure of the IDS, including the chronological sequence of the thematic sections (top) and the learning spirals addressed therein (bottom). The width of the boxes corresponds to the approximate relative time allocated to the thematic sections and learning spirals

In the spiral curriculum for biochemistry, usually several learning spirals converge in an individual class, e.g. in the IDS Joint Pain, which is part of the learning spiral nucleic acids. This IDS is taught in the module “Clinical Reasoning and Decision Making” in semester 7 as a corporate class with rheumatology and serves to achieve the biochemical learning objective “describe principles of the synthesis and degradation of nucleotides and derive and explain processes, symptoms and treatment options for nucleotide metabolism disorders using the example of gout”. The individual thematic sections of the biochemistry part cover six different learning spirals and the relative amount of time allocated to the content of the different learning spirals or thematic sections varies (see figure 4 (Fig. 4)).

## 3. Results

### 3.1. Comparative evaluation of learning success using the Progress Test Medicine (PTM)

We aimed to examine whether students in the BMM's spiral curriculum for biochemistry, which deviates from the traditional subject systematics in terms of content arrangement, achieve learning outcomes in biochemistry that are comparable to those of other curricula. Thus, in an accompanying scientific study, we analysed learning outcomes using the PTM [14] and compared them with those of students at the other participating universities.

#### 3.1.1. Analysis method and study population

The PTM is a formative assessment at graduate level [14]. Participation at the beginning of each semester is mandatory for BMM students. The PTM results from SS23, WS23/24 and SS24 were analysed. The majority of participating universities offer model degree programmes in accordance with the German ÄApprO (see attachment 3 A ). The number of participating students per semester and assessment ranged from 452 (SS23, semester 3) to 1848 (SS23, semester 4) (see attachment 3 B ).

#### 3.1.2. Statistical analysis

The statistics software R (version 4.4.3; R Core Team, 2025) was used for data management and statistical analyses. The normal distribution of the values was assessed by the Shapiro-Wilk test. Group differences were analysed using a t-test for normally distributed values in both compared groups (BMM and others), or using a Mann-Whitney U test for non-normally distributed values in one of the compared groups. Significant differences are assumed for p-values ≤0.05. The tests for non-inferiority were performed based on the t-test for normally distributed values or based on the Mann-Whitney U test for non-normally distributed values. Seven different equivalence thresholds Δ (Δ=Cohen’s d * SD_other_) were tested to investigate effect sizes from Cohen’s d=0.2 (small effects) to Cohen’s d=0.8 (large effects).

#### 3.1.3 Questions analysed

**Figure 5:**
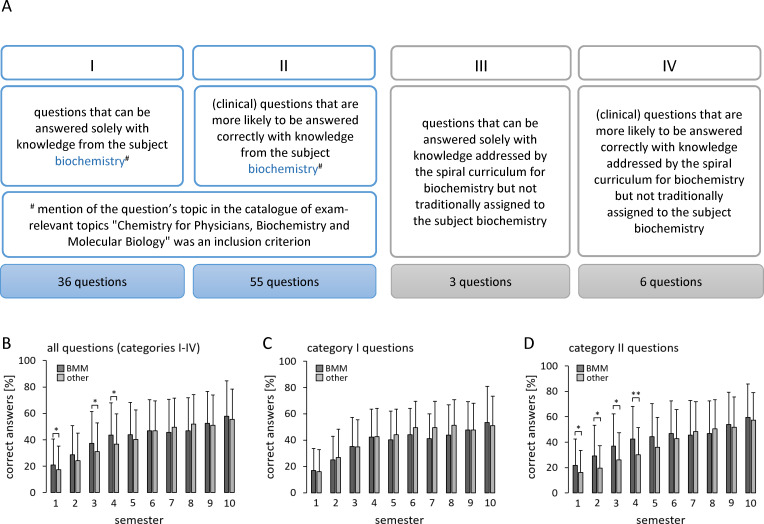
Comparative analysis of learning success in biochemistry using the Progress Test Medicine (PTM) A Definitions of categories I-IV, including the number of questions assigned from the PTMs of SS23, WS23/24 and SS24. B-D Mean±standard deviation of the proportion of PTM questions answered correctly by BMM students of semesters 1-10 compared with students from other universities participating in the PTM (see attachment 3). For statistical analysis, a t-test was performed for normally distributed data and a Mann-Whitney U test for non-normally distributed data. Significant differences are assumed for p-values ≤0.05. * indicates significant differences with a p-value of ≤0.05; ** indicates a significant difference with a p-value of ≤0.01

To select PTM questions for the analysis, we defined four categories for questions with biochemical content (see figure 5 A (Fig. 5)). Using a two-step approach, out of a total of 600 PTM questions we identified 101 questions and assigned them to one of the categories: In the first step, two members of the Institute of Biochemistry independently assessed whether the question corresponded to one of the categories. In the second step, all questions that were named by at least one member were assigned to one of the categories by consensus among at least three members or were assessed as not belonging to any of the categories. The mention of the question’s topic in the GK “Chemistry for Physicians, Biochemistry and Molecular Biology” was an inclusion criterion for category I or II, regardless of whether the topic was addressed in the spiral curriculum biochemistry (see figure 5 A (Fig. 5)).

#### 3.1.4. Research question 1: Do the BMM students’ PTM results in biochemistry differ significantly from other universities?

At the end of the study programme (semester 10) and in the majority of semesters, there was no significant difference in response behaviour between BMM students and students at the reference universities. In semesters 1, 3 and 4, BMM students answered significantly more questions correctly than students at the reference universities (see figure 5 B (Fig. 5)). An individual evaluation of the four categories showed that there were no significant differences in response behaviour for questions in category I, which tested isolated biochemical content (see figure 5 C (Fig. 5)). For questions in category II, in which biochemical knowledge was embedded in clinical contexts, BMM students in semesters 1-4 answered significantly more questions correctly compared to students at the reference universities (see figure 5 D (Fig. 5)). No significant differences were observed for questions in categories III and IV, although the statistical power for these categories is limited due to the small number of questions (3 and 6, respectively) (data not shown).

#### 3.1.5. Research question 2: Can it be ruled out that BMM students perform worse than students of other universities in answering biochemistry-related PTM questions?

Since the absence of evidence of a significant difference is not equivalent to evidence of non-inferiority, a non-inferiority test was performed. At the end of the study programme (semester 10), significant inferiority of BMM students compared to students at the reference universities could be ruled out with regard to questions in categories I-IV (small effects of Cohen’s d=0.2 with p≤0.05; medium and strong effects of Cohen’s d=0.5 - 0.8 with p≤0.001). Across all semesters, medium and strong effects could be ruled out (medium effects of Cohen’s d=0.6 with p≤0.05; strong effects of Cohen’s d=0.8 with p≤0.001).

Overall, we show that students in the spiral curriculum for biochemistry achieve at least equivalent learning success, and inferiority can be ruled out.

### 3.2. Evaluation of the spiral curriculum for biochemistry by BMM students

The spiral curriculum for biochemistry was evaluated by BMM students in semesters 1-10 using a 15-item questionnaire. Each item was rated on a 5-point Likert scale, with the additional option “I don’t know”. The questionnaire was piloted with a group of 4 students and adapted. The survey was conducted voluntarily and anonymously in the WS24/25 via the web application SoSci Survey [https://www.soscisurvey.de/]. By participating, the students agreed to the use of the results in a scientific study. No personal data was collected. No obligation for ethical consultation existed according to § 15 of the professional code of the Brandenburg State Medical Association (*Berufsordnung der Landesärztekammer Brandenburg*). Of the 574 students invited by email, 196 completed the questionnaire fully or in part (overall response rate 34.1%). Response rates per semester ranged from 49.3% (semester 3) to 21.6% (semester 8). The data was evaluated using descriptive statistics. The percentages for “strongly agree” and “agree” were combined as “agreement”.

**Figure 6:**
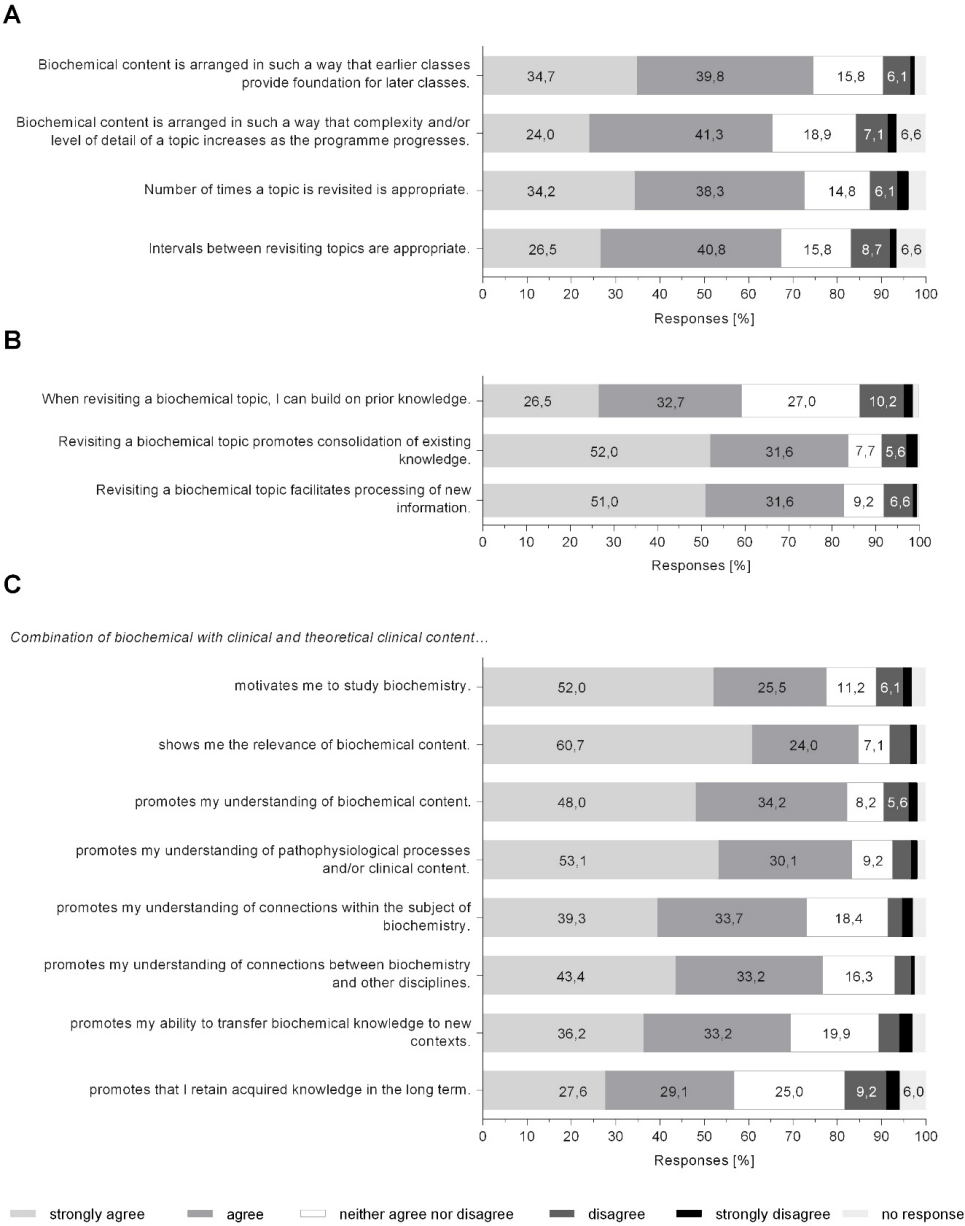
Evaluation of the spiral curriculum for biochemistry by BMM students Numbers correspond to percentages of all students of semesters 1-10 who completed the questionnaire fully or in part (n=196). A Evaluation of the curriculum structure, B Evaluation of the learning process, and C Impact of vertical integration. The survey was conducted using an electronic questionnaire comprising 15 items. Each item was rated on a 5-point Likert scale, with the additional option “I don’t know”

Regarding the structure of the spiral curriculum for biochemistry, the majority of students agreed that the biochemistry content was arranged in a way that earlier classes provide the foundation for later classes (74.5% agreement) and that the level of detail and complexity of a topic increased as the programme progressed (65.3% agreement). The majority of students rated the frequency and intervals for revisiting topics as appropriate (72.4% and 67.3% agreement) (see figure 6 A (Fig. 6)).

With regard to their learning process, the majority of students indicated that revisiting a topic allowed them to build on prior knowledge (59.2% agreement), consolidated existing knowledge (83.7% agreement) and made it easier for them to process new information (82.7% agreement) (see figure 6 B (Fig. 6)).

The majority of students supported that combining biochemical with clinical and theoretical clinical learning content demonstrates the relevance of biochemistry (84.7% agreement), motivates them to study biochemistry (77.6% agreement), and promotes their understanding of biochemistry and comprehension of connections within the subject biochemistry (82.1% and 73% agreement). The students also indicated that integration promoted their understanding of pathophysiological processes and clinical learning content (83.2% agreement) as well as the connections between biochemistry and clinical disciplines (76.5% agreement). In addition, the majority of students felt that integration promoted their ability to transfer biochemical knowledge to new contexts (69.4% agreement) and to retain acquired knowledge in the long term (56.6% agreement) (see figure 6 C (Fig. 6)).

## 4. Discussion

The spiral curriculum for biochemistry in the BMM meets the requirements specified in terms of subject systematics and provides an appropriate representation of the subject biochemistry in both breadth and depth. On the one hand, all learning spirals taken together show that the traditional biochemical topics are covered extensively in terms of breadth (see attachment 1 ). On the other hand, the exemplary learning spiral nucleic acids demonstrates depth of coverage by covering the relevant chapters of the GK and NKLM (see attachment 2 ). In addition, the majority of students state that earlier classes in the curriculum provide foundation for later classes (see figure 6 A (Fig. 6)), which suggests that the curriculum does not have any major gaps. This is also indicated by the at least equivalent learning success in the PTM (see figure 5 (Fig. 5)).

A characteristic feature of the spiral curriculum for biochemistry is that it possesses a relatively small number of TH. Its total number of 148 is substantially lower than that of other medical study programmes, which we have derived from five randomly selected, publicly available study regulations (304 to 397 TH for the subjects of chemistry, biochemistry and molecular biology) [15], [16], [17], [18], [19]. This is a consequence of didactic reduction [20] that takes place in the BMM on three levels: “tailoring to the level of knowledge in the early stages of the study programme”, “focusing on essential concepts with exemplary learning” and “reducing the number of topics in individual classes”. At the same time, PBL is the central teaching/learning format and students work on biochemical learning objectives in PBL-guided self-study without this being reflected in the TH [4]. Another feature of the spiral curriculum for biochemistry is that subject boundaries may shift, e.g. in the learning spiral nucleic acids between biochemistry and biology or pharmacology (see attachment 2 ). Such shifts occur several times in the curriculum. They are explicit assignments of responsibility made during interdisciplinary module planning conferences and apply to learning content at the intersection of several subjects.

One risk of spiral curricula expressed by critics is that dividing the subject into various, small content units could result in a loss of internal logic, making it difficult for students to recognise subject-inherent connections [2], [10]. The example of the nucleic acids learning spiral shows that the spiral curriculum does follow an internal logic and is structured from simple to complex and more detailed learning content (see attachment 2 ). From the students' perspective, the internal logic is reflected, for instance, in a functional structure and the development of the learning material from simple to complex (see figure 6 A (Fig. 6)). In addition, the majority of students regards the revisiting of biochemical topics positively, particularly in respect to characteristic elements of learning spirals, namely the activation and consolidation of prior knowledge as well as its connection to new information [21] (see figure 6 B (Fig. 6)). The repeated engagement with content is a typical feature of spiral curricula and provides the basis for students to derive broader basic science concepts and general principles [5]. The students' perception that both their understanding of biochemical content and their ability to identify subject-inherent connections are promoted (see figure 6 C (Fig. 6)) suggests that the subject of biochemistry is accessible for students due to its comprehensible internal logic. This perception is confirmed by the students' learning success in biochemistry, which is at least equivalent to that of students in other model and traditional study programmes (see figure 5 (Fig. 5)).

The degree of curricular integration can be evaluated using the integration ladder developed by Harden [22], [23], [24]. This defines 11 levels: from isolation (subject-specific teaching without awareness of the other subjects’ teaching content) to transdisciplinarity (complete dissolution of subject boundaries and immersion in the clinical learning environment). The spiral curriculum for biochemistry in the BMM can be classified as level 9, multidisciplinarity [24]. The prerequisite for this high degree of integration is created by the structure of the BMM, in particular by the organ- and topic-related modules, the central importance of PBL and the resulting focus of the learning process on interdisciplinary problems. The decisive factor for classification at level 9 is the complete integration of the spiral curriculum for biochemistry throughout the entire length of the study programme and across all structural levels. Biochemistry views topics through its subject’s lens and contributes to the students’ understanding through the biochemical perspective. While the subject identity is retained, subject autonomy is largely abandoned [24]. This is evident, for example, in examinations, which take place at the end of each semester as joint interdisciplinary exams across several modules. According to the students' perception, vertical integration promotes understanding of clinical learning content and the connections between biochemistry and other subjects (see figure 6 C (Fig. 6)). Furthermore, vertical integration highlights the relevance of biochemical learning content and has a motivating effect (see figure 6 C (Fig. 6)). This demonstrates the successful implementation of the principles of vertical integration and confirms the opportunities associated with its use.

### 4.1. Limitations

One limitation of the accompanying study is that, due to the relatively low number of students in the BMM, the survey was conducted with students from all semesters. Given that students in the early study phase have not yet completed the entire curriculum, it cannot be ruled out, that this affects the evaluation. Naturally students are, apart from the BMM, not familiar with any other biochemical curriculum and therefore unable to compare its structure and effects, which limits the significance of the results. In addition to the systematics of the spiral curriculum for biochemistry of the BMM, students come into contact with the traditional subject systematics of biochemistry through textbooks and other learning resources. The accompanying study was not designed to investigate the effect of the spiral curriculum’s subject systematics on learning behaviour and success under controlled conditions, i.e. free from the influences of other subject systematics. The findings are therefore limited to the effects of the subject systematics of the spiral curriculum in the real world learning environment. 

Limitations of the BMM’s spiral curriculum include the considerable time required for consultation and coordination between the individual disciplines and the difficulty of incorporating thematic advancements and new scientific findings into the curriculum [25]. In addition, the various learning spirals in the BMM's spiral curriculum for biochemistry vary in size, and it cannot be ruled out that the expansion of individual learning spirals, such as biochemical laboratory techniques, would have a positive effect on the students' overall understanding.

## 5. Conclusion

With the spiral curriculum for biochemistry in the BMM, we have developed and successfully implemented a curriculum that implements the central recommendations for the further improvement of basic science medical education, as stated by the German Science and Humanities Council [1] and the drafts for the amendment of the ÄApprO of 2020 and 2023 [26], [27].

Regardless of the current uncertainty in respect to content and timing of the amendment of the ÄApprO, we would like to use this specific example to foster the discussion on the further improvement of medical education, in particular by strengthening vertical integration, and to support curricular development processes both in medical education and in other health professions.

## Notes

### Use of AI-supported programmes

DeepL Pro (DeepL AI GmbH) was used for the first draft of the English translation of the manuscript. The draft was subsequently reviewed and revised by the authors, who take full responsibility for the content.

### Authors’ ORCIDs

- Jenny Engelmann: [0009-0004-4705-0037]
- Julia Schendzielorz: [0000-0003-2471-094X]
- Fabian Otte: [0009-0008-3068-5967]
- Meike Hoffmeister: [0000-0003-3561-5286]
- Stefanie Oess: [0000-0001-7381-1216]

## Acknowledgements

We thank Andreas Winkelmann (Institute of Anatomy, MHB) for his conceptual support in assessing learning success, Maren März and Iván Roselló Atanet (Charité, Berlin) for providing the original PTM data and their support in the analysis, and Luca Caramenti (Institute of Biostatistics and Registry Research/Centre for Clinical Studies, MHB) for statistical support. We would also like to thank the student assistants/doctoral candidates at the Institute of Biochemistry, in particular Vivien Latuske and Ronny Gierecki, for piloting the questionnaire. We thank Steve Swendeman (Boston) for help with the English translation.

## Competing interests

The authors declare that they have no competing interests. 

## Supplementary Material

Learning spirals within the spiral curriculum for biochemistry in the BMM

Learning spiral of nucleic acids

Participation in the Progress Test Medicine (PTM)
