# Supplementary material for: Vertical integration of biochemistry: The interdisciplinary spiral curriculum in the Brandenburg reformed medical study programme
Source: GMS J Med Educ. 2026 Jun 15;43(5):Doc63. doi: 10.3205/zma001857 (PMC13316365; doi:10.3205/zma001857)
Supplement: Learning spirals within the spiral curriculum for biochemistry in the BMM [file JME-43-63-s-001.pdf]

## Attachment 1: Learning spirals within the spiral curriculum for biochemistry in the BMM

Learning spirals within the spiral curriculum for biochemistry, their distribution across modules and number of assigned learning objectives. The learning objectives were assigned to individual classes by the respective teachers and subsequently aggregated for each module. The individual learning objectives vary in granularity, and their number does not correlate directly with teaching hours or number of content units.

| Learning spirals | | matter | chemical reactions | nucleotides | nucleic acids | carbohydrates | lipids | proteins | enzymes | cytoskeleton | extracellular matrix | signalling | cell cycle/ cell death | energy metabolism | biochemical laboratory | gastrointestinal tract | liver | kidney | blood | immune system | muscles | bones, cartilage | skin | nervous system |
|---|---|---|---|---|---|---|---|---|---|---|---|---|---|---|---|---|---|---|---|---|---|---|---|---|
| Sem | Module | | | | | | | | | | | | | | | | | | | | | | | |
| 1 | Movement | 4 | 4 | 1 | 1 | 2 | 1 | 3 | 2 | | 3 | 2 | | 1 | | 1 | | 1 | | | | 2 | | 1 |
| | Cardiovascular System | | 3 | | 1 | | 3 | 3 | 3 | | | 1 | | 4 | | 3 | 2 | | 1 | | | | | |
| 2 | Respiration | 1 | 1 | | | | | | | | | | | | | | | | | | | | | |
| | Blood | 3 | 3 | 2 | 3 | 2 | 2 | 5 | 3 | 2 | 2 | 2 | 1 | 2 | 1 | 1 | 1 | | 8 | 1 | | | | |
| | Nutrition/Digestion/ Metabolism | | 2 | 2 | | 6 | 7 | 6 | 5 | | | 5 | | 6 | | 3 | 6 | 2 | 2 | | 3 | | | |
| 3 | Nervous System | | | 1 | | | 1 | 2 | 1 | | | 2 | | | | | | | | | | | | 2 |
| | Inflammation/Immune Response | 3 | 2 | 3 | 6 | | 7 | 14 | 8 | 1 | 1 | 6 | 7 | | 3 | | | 1 | 2 | 10 | | | 1 | |
| 4 | Kidney | | 2 | | | 1 | 2 | 2 | 1 | | | 2 | | 1 | | 2 | 1 | 4 | 4 | | | 1 | 1 | |
| | Experience and Behaviour | | | | | 2 | 2 | 1 | | | | 2 | | 2 | | 2 | 2 | | 2 | 2 | 2 | | | 2 |
| 5 | Hormones/Sexual Organs/Sexuality | 2 | 3 | 2 | 5 | 4 | 5 | 5 | 3 | 1 | | 9 | 8 | 4 | 1 | 4 | 4 | 3 | 5 | 1 | 5 | 5 | 5 | 6 |
| 7 | Clinical Reasoning and Decision Making | | | 1 | 1 | | 1 | | 1 | | | | 1 | | | 1 | 1 | 1 | 1 | | | 1 | 1 | |
| 8 | Obstetrics and Gynaecology | | | | 1 | | | 1 | | | | 2 | 3 | | | | | | 1 | 1 | | 1 | 1 | 2 |
| | Paediatrics | | | | 1 | 2 | 1 | 1 | 2 | | | 2 | 1 | 1 | | | 1 | 1 | 3 | 1 | | | 1 | |
| 10 | Geriatrics | | | | 1 | 1 | 1 | 1 | | | | 1 | 1 | 1 | | | | | | 1 | | | | |

Sem: Semester

Attachment 1 to Engelmann J, Schendzielorz J, Otte F, Bertram A, Hoffmeister M, Oess S. *Vertical integration of biochemistry: The interdisciplinary spiral curriculum in the Brandenburg reformed medical study programme*. GMS J Med Educ. 2026;43(5):Doc63. DOI: 10.3205/zma001857
