# Supplementary material for: Vertical integration of biochemistry: The interdisciplinary spiral curriculum in the Brandenburg reformed medical study programme
Source: GMS J Med Educ. 2026 Jun 15;43(5):Doc63. doi: 10.3205/zma001857 (PMC13316365; doi:10.3205/zma001857)
Supplement: Learning spiral of nucleic acids [file JME-43-63-s-002.pdf]

Attachment 2: Learning spiral of nucleic acids

Distribution of the topic nucleic acids over individual content units (highlighted in grey), their chronological sequence across semesters and modules; reference to PBL case and weekly topic; class format in which the content unit is mainly addressed; for IDS, the discipline of the co-teacher; specification of the module-specific (sub)topic or class focus in which the biochemical content unit is integrated; related modules' learning objective; assigned NKLM and GK content.
* Indicates PBL cases or weekly topics directly related to the biochemical content unit; if no PBL case/weekly topic is specified, the class follows the inverted classroom principle with asynchronous components not allocated to a particular module week. [1] In semester 7, two PBL cases are assigned per week.

Sem	Module BMM	PBL case/ Weekly topic	Biochemical content unit	Class format	Co-discipline (in IDS)	Integration with topic	Modules' learning objective *Students should be able to...*	NKLM content	GK
1	Movement	-	Structure of nucleic acids (chemical basics)	LP/T	-	-	describe the structure of DNA and RNA as well as the basic principles of replication, transcription and translation	VII.1a-02.3.6	12.1.1 12.1.2
		Fracture of the femoral neck (osteoporosis)/ Bone metabolism*	Structure and properties of nucleic acids	IDS	Anatomy	Structure and function of osteoblasts and osteoclasts		VII.1a-02.3.1 VII.1a-02.3.6 VII.1a-02.5.3	12.1.1 12.1.2 12.2.1 19.2.1
			Basic principles of replication and transcription						
		Supination trauma/ Connective tissue*	Basic principles of translation	IDS	Anatomy	Collagen biosynthesis		VII.1a-02.5.7	19.2.1
	Cardio-vascular System	Peripheral arterial occlusive disease (PAOD)/ Arteriosclerosis*	Principles of regulated gene expression	IDS	Cardiology	Cholesterol biosynthesis, in particular regulation of HMG-CoA reductase quantity	describe the principles of enzyme activity regulation using the example of HMG-CoA reductase	VII.1a-02.5.7 VII.1a-02.4.3	15.5 19.2.1
2	Blood	(Iron deficiency) anaemia/ Blood composition and function*	Mechanisms of gene expression regulation	IDS	Haematology	Regulation of haem synthesis by EPO and iron (Fe^{2+})	describe important events in erythropoiesis and their regulation, in particular by erythropoietin	VII.1a-02.5.7	19.2.5 25.1.4
		(Iron deficiency) anaemia/ Blood composition and function*	Fundamentals of nucleotide biosynthesis	IDS	Haematology	Function of tetrahydrofolate and vitamin B_{12} in nucleotide synthesis, including significance for pernicious anaemia	describe the basic principles of iron, vitamin B_{12} and folic acid metabolism and name the causes of their deficiency	VII.1a-02.3.6 VII.1a-06.2.6	19.1.1 13.4
		Bleeding complications during phenprocoumon therapy/ Haemostasis and transfusion medicine*	Principles of heredity	IDS	Haematology/ Transfusion medicine	Blood types and their inheritance	explain the basic principles of clinically relevant blood type genetics and blood type serology	VII.1a-02.5.5	2.3 GK-Bio

3	Inflammation/<br>Immune Response	-	Genome organisation, replication, transcription, translation and regulation of gene expression in pro- and eukaryotes, in comparison	LP/T	-	-	describe the structure of chromosomes and genes, explain the storage of information in nucleic acids and describe characteristic differences between eukaryotes, prokaryotes and viruses;<br>explain the replication of genetic information, transcription, RNA modification and translation, as well as their regulation and describe characteristic differences between eukaryotes, prokaryotes and viruses	VII.1a-02.5.1<br>VII.1a-02.5.2<br>VII.1a-02.5.3	19.2.2
				LP/T	-	-		VII.1a-02.5.7	19.2.4<br>19.2.6
				LP/T	-	-		VII.1a-02.5.7	19.2.7<br>19.2.8
				LP/T	-	-		VII.1a-02.5.7	19.2.5<br>19.2.9<br>19.2.11
		-	Basic principles of DNA mutation and repair	LP/T	-	-	describe the mechanisms of DNA mutation and repair;<br>explain the principles of heredity and evolution	VII.1a-02.5.4<br>VII.1a-02.5.5	19.2.3<br>2.9 GK-Bio
			Mechanisms of genetic evolution						
		Overwhelming post-splenectomy infection syndrome (OPSI)/<br>specific immune system, B lymphocytes*	Somatic recombination	IDS	Immunology	Production of antibody diversity, isotype switch	describe the structure of chromosomes and genes, explain the storage of information in nucleic acids, and describe characteristic differences between eukaryotes, prokaryotes, and viruses;<br>describe the function, classification and biosynthesis of immunoglobulins	VII.1a-02.5.1<br>VII.1a-02.5.2	19.2.1<br>24.3
			Transcription and translation			Immunoglobulin synthesis		VII.1a-02.5.7	
		Overwhelming post-splenectomy infection syndrome (OPSI)/<br>specific immune system, B lymphocytes	Replication, transcription in molecular detail	P	-	Nucleoside analogues, *lac* operon, cap snatching	describe the structure of chromosomes and genes, explain the storage of information in nucleic acids, and describe characteristic differences between eukaryotes, prokaryotes, and viruses;<br>explain the replication of genetic information, transcription, RNA modification and translation, as well as their regulation, and describe characteristic differences between eukaryotes, prokaryotes and viruses;<br>explain the mechanisms of antibiotic resistance development and the significance of antibiotic resistences	VII.1a-02.3.6<br>VII.1a-02.5.3	12.2.1<br>19.2.2<br>19.2.4<br>19.2.6
		Needlestick injury/<br>Viral infections*	Translation in molecular detail	EDT	-	*CCR5Δ32*-mediated HIV resistance		VII.1a-02.5.7	19.2.7<br>19.2.10
		Multiresistant germs/<br>Antibiotic therapy and resistance development*	Replication, transcription, translation in molecular detail	IDS	Pharmacology	Topoisomerase and ribosome as targets for antibiotic therapy		VII.1a-02.5.3<br>VII.1a-02.5.7<br>VII.1a-02.5.5	19.2.2<br>19.2.4<br>19.2.8<br>3.5 GK-Bio
			Antibiotic resistance through mutation			Antibiotic resistance			

5	Hormones/ Reproductive Organs/ Sexuality	Hyperthyroidism/ Hypothalamic-pituitary-thyroid axis*	Molecular mechanisms of regulated gene expression, transcription factors as receptors for lipophilic hormones	IDS	Endocrinology	Hypothalamic-pituitary-target organ axes	explain the principles of cellular signal transduction mechanisms of hormones and their effects at the cellular level;<br>describe the principles of hydrophilic and lipophilic hormone action using the example of the hormones of the hypothalamus, the pituitary gland and their target organs	VII.1a-02.5.7	19.2.5<br>23.1.3
		Addison's disease/ Hypothalamic-pituitary-adrenal axis*		IDS	Endocrinology				
		-		LP/T	-				
		Gonorrhoea/ sexually transmitted infections		P	-	Effect of thyroid hormones on gene expression in cardiomyocytes		VII.1a-02.5.7	19.2.5<br>23.2.6
		-	Mitosis, meiosis	LP/T	-		apply the basic concepts of cell division and explain chromosomal abnormalities	VII.1a-02.5.6<br>VII.1a-03.3.2	20.11<br>1.14 GK-Bio<br>1.15 GK-Bio
		Androgen insensitivity/<br>Sex development*	Numerical chromosome aberrations	IDS	Gynaecology	Chromosomal sex determination, Turner syndrome, Klinefelter syndrome	apply the basic concepts of cell division and explain chromosomal abnormalities;<br>explain the genetic and hormonal principles of sexual differentiation	VII.1a-02.5.1<br>VII.1a-02.5.2	2.4 GK-Bio<br>23.1.3
7	Clinical Reasoning and Decision Making	Gout and rhizarthrosis[1]/<br>Joint pain*	Nucleotide metabolism, synthesis and degradation of nucleotides, *salvage pathway*	IDS	Rheuma-tology	Gout	describe principles of the synthesis and degradation of nucleotides and derive and explain processes, symptoms and treatment options for nucleotide metabolism disorders using the example of gout	VII.1a-06.1.10<br>VII.1a-06.2.6<br>VII.1b-03.15.8	19.1.1<br>19.1.3
8	Obstetrics and Gynaecology	Breast cancer/ gynaecological malignancies*	Cell cycle, oncogenes, tumour suppressors, viral oncogenes	IDS	Gynaecology	HPV infection, genital carcinoma	name aetiology (including molecular/cellular/genetic principles of tumorigenesis), symptoms (esp. key symptoms), diagnostics, rough classification, basic principles of stage-appropriate therapy, as well as complications of gynaecological malignancies using the example of vulvar, cervical, corpus, breast and ovarian carcinoma	VII.1a-02.5.4<br>VII.1a-03.3.1	19.2.3<br>19.2.10<br>19.5.1<br>20.11.1
8	Paediatrics	Acute leukaemia/ Paediatric oncological diseases*	DNA mutation and repair, telomerase	L	-	Multi-step process of tumour development	explain common and important chronic diseases in child and youth medicine, including symptoms, pathophysiology, diagnostics and basic therapeutic measures, as well as psychosocial complications: […]<br>b. blood disorders/oncological diseases […]	VII.1a-02.5.4<br>VII.1a-03.3.1	12.2.2<br>19.2.2<br>19.2.3<br>19.5.1

Attachment 2 to Engelmann J, Schendzielorz J, Otte F, Bertram A, Hoffmeister M, Oess S. *Vertical integration of biochemistry: The interdisciplinary spiral curriculum in the Brandenburg reformed medical study programme*. GMS J Med Educ. 2026;43(5):Doc63. DOI: 10.3205/zma001857

10	Geriatrics	Polypharmacy/ Multimorbidity in old age*	Genetic instability, epigenetic changes, replicative senescence	L	-	Biochemistry of ageing	explain the biochemical aspects of ageing	VII.1a-02.5.7	19.2.1<br>19.2.5

EDT: Exercises in Diagnostics and Therapy; GK: Catalogue of Exam-relevant Topics (Subcatalogue "Chemistry for Physicians and Biochemistry/Molecular Biology", January 2014) of the Institute for Medical and Pharmaceutical Examination Questions; GK-Bio: Catalogue of Exam-relevant Topics (Subcatalogue "Biology for Physicians", January 2014) of the Institute for Medical and Pharmaceutical Examination Questions; IDS: Interdisciplinary Seminar; L: Lecture; LP/T: Learning Platform/Tutorial; NKLM: National Competence Based Catalogue of Learning Objectives for Undergraduate Medical Education 2.0, as of March 2025; P: Practical Class; PBL: Problem-Based Learning; Sem: Semester
