# Supplementary material for: Vertical integration of biochemistry: The interdisciplinary spiral curriculum in the Brandenburg reformed medical study programme
Source: GMS J Med Educ. 2026 Jun 15;43(5):Doc63. doi: 10.3205/zma001857 (PMC13316365; doi:10.3205/zma001857)
Supplement: Participation in the Progress Test Medicine (PTM) [file JME-43-63-s-003.pdf]

Attachment 3: Participation in the Progress Test Medicine (PTM)

A Number of all participating universities in the PTM of SS23 (PTM 48), WS23/24 (PTM 49) and SS24 (PTM 50), including the Brandenburg Medical School, indicating proportions of German, Austrian and Swiss universities and the type of study programme, respectively.
B Number of serious participants in the PTM of SS23 (PTM 48), WS23/24 (PTM 49) and SS24 (PTM 50) from the BMM and other universities per semester.

**A**	PTM	SS23	WS23/24	SS24
	total	17	14	15
	from Germany	12	10	11
	thereof standard degree programme	2	2	2
	thereof model degree programme	10	8	9
	from Austria	4	3	3
	from Switzerland	1	1	1

**B**	PTM	SS23		WS23/24		SS24	
	Semester	BMM	other	BMM	other	BMM	other
	1	61	423	50	948	63	467
	2	46	968	48	522	48	918
	3	35	417	38	1642	56	429
	4	41	1807	42	432	40	1107
	5	42	453	43	1086	39	460
	6	38	1647	42	531	40	1052
	7	43	534	39	1313	45	536
	8	24	982	42	650	40	1121
	9	44	424	24	882	43	502
	10	0	1475	41	515	25	857

Attachment 3 to Engelmann J, Schendzielorz J, Otte F, Bertram A, Hoffmeister M, Oess S. *Vertical integration of biochemistry: The interdisciplinary spiral curriculum in the Brandenburg reformed medical study programme*. GMS J Med Educ. 2026;43(5):Doc63. DOI: 10.3205/zma001857
